# How Leader-Member Exchange Affects Creative Performance: An Examination From the Perspective of Self-Determination Theory

**DOI:** 10.3389/fpsyg.2020.573793

**Published:** 2020-10-28

**Authors:** Zhiyu Xie, Na Wu, Tong Yue, Jing Jie, Guanghui Hou, Anguo Fu

**Affiliations:** ^1^School of Management, Hainan Institute of Corporate Governance, Hainan University, Haikou, China; ^2^Faculty of Psychology, Research Center for Psychology and Social Development, Southwest University, Chongqing, China; ^3^Department of Law, Shantou University, Shantou, China

**Keywords:** leader-member exchange, intrinsic motivation, positive moods, creative performance, self-determination theory

## Abstract

It has been shown that leadership is a major factor that influences creative performance. Although past studies have found that leader-member exchange (LMX) has direct effects on employee creative performance, there continues to be a lack of research examining how the LMX relationship mediates creative performance. This study used self-determination theory to examine the mediating effects of the LMX relationship on creative performance through attitudinal and emotional processes. Participants were supervisors and subordinates of township enterprises in the Pearl River Delta in China. There were 386 valid supervisor-subordinate dyads. Supervisors were responsible for assessing creative performance and the remaining variables were completed by employees. Results showed that high LMX increased the positive moods of subordinates, improved creative performance, and stimulated intrinsic motivation for improvement. Based on the results, we have proposed academic and practical recommendations such as organizations that want to encourage creativity, could begin by training managers to demonstrate high LMX by strengthening their relationships with employees. We also described study limitations, and suggested directions for future studies.

## Introduction

In the context of increasingly fierce competition among enterprises, employee creativity is an important factor in determining company survival and growth. Therefore, examining the factors that affect creative performance is an important academic and managerial issue.

Previous studies have classified variables that affect employee creativity into those related to individual differences and organizational situations. Individual-difference variables include negative/positive moods (George and Zhou, 2007; To et al., 2015; Han et al., 2019), personality traits, such as openness to experience and conscientiousness (George and Zhou, 2001), and core self-evaluation (Saeed et al., 2019). Organization-related variables include work characteristics such as work complexity (Shalley et al., 2004), person-organization fit (Seong and Choi, 2019), and leadership (Tierney et al., 1999; Shin and Zhou, 2003; Wang and Cheng, 2010).

Among leadership-related variables, previous studies have examined the effects of transformational leadership (Shin and Zhou, 2003; Gong et al., 2009), shared leadership (Sun et al., 2016), benevolent leadership (a form of Chinese paternalistic leadership) (Wang and Cheng, 2010), moral leadership (Gu et al., 2015), and humble leadership (Wang et al., 2018) on the creative performance of employees. However, these studies primarily focused on the perceptions of top-down leadership and did not consider interaction rules between managers and employees. In contrast, leader-member exchange (LMX) encompasses the interactive relationship between managers and employees and represents a social exchange process ranging from low to high exchange. Previous studies have highlighted that the quality of interpersonal exchange in LMX affects employee creativity (Volmer et al., 2011; Saeed et al., 2019). However, there remains a lack of research on the mediators of the relationship between LMX and creativity (Atwater and Carmeli, 2009; Liao et al., 2010; Pan et al., 2012; Carnevale et al., 2017; Seong and Choi, 2019). Atwater and Carmeli (2009) recommended that to examine the complex relationship between leadership and creativity, future studies should clarify possible mediating mechanisms between LMX and creativity. Accordingly, the current study used the paired relationship quality (LMX) between managers and employees as a main axis by which to examine possible mediating mechanisms between LMX and employee creativity.

This study used self-determination theory as proposed by Ryan and Deci (2000) as a basis to explain the mediating processes between LMX and creativity. Based on this theory, when external situational factors (such as LMX) satisfy three individual needs (competence, autonomy, and relatedness), they will not only aid individual psychological growth (such as intrinsic motivation), but also affect psychological health (such as positive moods), thereby affecting individual behavior (Reis et al., 2000; Ryan and Deci, 2000, 2001; Baard et al., 2004). Therefore, this study first explains how LMX can induce, promote, and satisfy competence, autonomy, and relatedness. While examining the mediating process between LMX and creative performance, self-determination theory has been used as a basis to explain how intrinsic motivation is developed, and positive moods are produced from the followers’ attitudinal (such as intrinsic motivation) and emotional (such as positive moods) processes (i.e., from the fulfillment of the needs of competence, autonomy, and relatedness) to discuss possible mediating mechanisms.

Regarding attitudinal processes, previous studies have noted that as creativity usually involves risk and uncertainty, an individual’s intrinsic motivation is usually the driving force behind creative output (Amabile, 1983; Zhang and Bartol, 2010). Therefore, our study considers intrinsic motivation as a mediating attitudinal mechanism between LMX and creative performance.

Regarding emotional processes, Amabile’s (1983) suggests the following with regard to the “componential conceptualization” of creativity (as cited by Zhang and Bartol, 2010, p. 108): “Intrinsic task motivation is a necessary, but not sufficient, condition for creative outcomes.” Thus, if employees are unable to link a problem with their own knowledge structure when judging problems, they will be unable to use cognitive complexity and elasticity to produce creative ideas (Baas et al., 2008). Many studies have found that people with positive moods exhibit thought processes that are beneficial to producing creativity, such as divergent and fluent thinking (George and Zhou, 2007; To et al., 2015; Han et al., 2019). Similarly, people who feel joyful show greater ideational fluency (Abele-Brehm, 1992; Xiao et al., 2015). Therefore, our study considers positive mood as a mediating emotional mechanism between LMX and creative performance.

In summary, our study uses attitudinal processes (such as intrinsic motivation) and emotional processes (such as positive moods) to explain the mediating mechanism between LMX and creative performance. Self-determination theory was used as a framework to examine the effect of LMX on competence, autonomy, and relatedness in employees, as well as on intrinsic motivation and positive mood, which lead to increased creative output. Our study elaborates on the self-determination theory to explain how external factors (such as LMX) can develop intrinsic motivation and increase positive moods (the antecedents), and further expands it to individual behavioral performance (the outcomes). At the same time, we integrated the effects of LMX on creative performance through emotional and attitudinal mechanisms. This provides a meaningful perspective for further understanding the relationship between LMX and creative performance.

### Literature Review and Research Hypothesis

To examine leadership relationships, LMX uses interactions between leaders and followers. Due to limited resources, leaders and followers establish different degrees of leadership relationships (Graen and Uhl-Bien, 1995). Further, LMX is a form of reciprocal behavior based on social exchange theory. High LMX provides followers with tangible and psychological support. Through tangible support, managers can provide additional work-related information resources to employees, non-formal feedback and guidance, and participation in decision-making. Through psychological support, managers can provide supportive expressions, trust, respect, and autonomy. Therefore, with high LMX, employees are willing to invest more effort and take on more responsibility.

Blau’s (1964) social exchange theory notes that during interpersonal interactions, people can produce unclear future obligations. When one party provides a favor to the other, the former will expect possible future returns. However, the time and form of the return are not clear (Gouldner, 1960). Both parties will develop trust, responsibility, and gratitude based on reciprocal results in social exchange. Hence, with high LMX, a manager will provide support, trust, and respect and like their employees (Schriesheim et al., 1999). Furthermore, managers will give their employees autonomy to make decisions (Rosse and Kraut, 1983; Scandura et al., 1986), and more resources (Rosse and Kraut, 1983; Dunegan et al., 1992). Therefore, employees will repay managers with better performance.

Previous research has shown that the effects of LMX on creative performance include two aspects: emotions and supportive work resources. Regarding emotions, a high exchange relationship can provide an atmosphere of ease and trust for employees. High LMX indicates that the relationship between managers and employees is similar to a partnership. Both parties reciprocate, and there is mutual trust, respect, and liking. These traits promote an atmosphere of ease and trust (Schriesheim et al., 1999; Gu et al., 2015). In addition, the implementation of creativity sometimes requires a deviation from the status quo, challenging traditional methods, or adopting non-habitual behavior, which requires employees to bear the risk of possible failure (Berg et al., 2017). Therefore, high LMX can enable followers to be more comfortable engaging in non-traditional behavior, generating innovative ideas, and breaking away from habits, which aids creativity (Mumford and Gustafson, 1988; Carnevale et al., 2017).

Regarding supportive work resources, employees with high LMX will usually have more opportunities to participate in challenging tasks and obtain more resources, such as information and feedback (Dunegan et al., 1992; Carnevale et al., 2017). Therefore, compared with employees with low LMX, those with high LMX have a greater degree of empowerment and development. They will be more inclined to accept challenges and willing to accept the risk of failure. This also aids the production of creative performance (Graen and Uhl-Bien, 1995; Berg et al., 2017).

In summary, with high-quality LMX, managers are willing to support their employees and there is mutual trust, respect, and liking. This creates a relaxed and enjoyable work environment. Furthermore, managers provide additional resources, thereby enabling employees to accept challenging tasks, and provide supportive or work-related feedback to assist employees in generating creative output. Thus, employees that feel empowered and valued at work, are more willing to contribute ideas and opinions, and have the confidence to undertake challenging tasks and bear risks, which enables them to further demonstrate creative performance.

### Mediating Effects of Intrinsic Motivation

Intrinsic motivation refers to the attraction to the value of a task itself. This is associated with a driven state of motivation, which is a type of endogenous psychological need. When a person has high intrinsic motivation, they will seek and accept the most suitable new challenges (Deci and Ryan, 1985).

Ryan and Deci (2000) defined “self-determination” as the ability to choose one’s own behavior without the influence of external stress or obligations. Further, they stated that interactions among individuals and the social environment are the foundation of individual behavior, experiences, and intrinsic motivation. The external environment (such as communication, feedback, and reward) satisfies three individual needs, namely competence, autonomy, and relatedness, enabling individuals to develop behavioral motives with different degrees of self-determination. When self-determination of behavior is high, the probability that this behavior becomes part of the self is also high. Competence refers to the individual’s perception that he or she can complete challenging tasks through interaction with the social environment. In addition, individuals can predict that these activities will increase their capabilities. Autonomy refers to the concept that individuals control their own behaviors, believing that behaviors originate from self-volition and not stress, obligation, or other external pressure. Relatedness refers to the individual’s ability to establish respect, trust, care, and safety with others. Overall, competence and autonomy are associated with the individual’s work-related behaviors, while relatedness is a driving force underlying interpersonal relationships. Therefore, the most important aspect of self-determination theory is that an individual’s intrinsic motivation can be cultivated through the external environment and incentives. When external incentives are present or exist in situations that include specific behavioral development factors, even if an individual is not interested in certain behaviors at first, they will internalize, integrate, and gradually regulate these behaviors over time. Even if an individual initially adopts a certain behavior because of external situational factors, if an important person or group encourages that individual to engage in such behavior, the individual will internalize and integrate the behavior. The higher the degree of integration and internalization, the greater the degree of motivational self-determination for the individual to partake in such behaviors.

Thus, there are situations that support competence, autonomy, and relatedness in individuals (such as high LMX) and cause them to perceive that they can control and be responsible for a task’s process or results. Furthermore, such situations encourage the individual to feel that they are valued and loved and possess security and value. These situational factors cause individuals to develop intrinsic motivation, which aids in improving creativity. Accordingly, we infer that intrinsic motivation is a mediating mechanism between LMX and creative performance.

First, with high LMX, it is easier for a manager to understand employee problems (Carnevale et al., 2017) and provide resources and authority to solve work-related problems. Compared with employees with low LMX, those with high LMX receive more work-related feedback and guidance (Lam et al., 2017), which helps to improve their work competence. Graen and Cashman (1975) also noted that followers with high LMX have more opportunities to participate in challenging tasks.

Second, high LMX also assists in satisfying employees’ need for autonomy. Scandura et al. (1986) suggested that employees with high LMX experience greater work participation and perceived importance. In addition, these employees will provide ideas or assistance that improve workplace efficiency. Furthermore, the manager will provide interpersonal care and support to employees, which helps them to accept the manager’s leadership and internalize and integrate the behaviors encouraged by the manager. This encouragement then becomes the intrinsic motivation for employees to engage in these behaviors.

Therefore, when followers can satisfy their competence, autonomy, and relatedness needs through high LMX with their leaders, their intrinsic motivation is stimulated, and they develop greater interest and enthusiasm for their work. At this point, employees can actively complete tasks and generate better ideas or search for better methods, thereby improving their creative performance.

Based on the above, we propose the following hypotheses:

**H1**: Intrinsic motivation is a mediating mechanism between LMX and creative performance. That is, high LMX will stimulate intrinsic motivation in employees, thereby encouraging them to exhibit greater creative performance.

### Mediating Effects of Positive Mood

Mood is a pervasive and generalized affective state (George and Zhou, 2007) in which transient emotional expressions persist longer than emotions do and fluctuate because of situational factors and interactions (George, 1991). Specifically, moods are different from emotions because they do not have specific and clear targets or causes (Baas et al., 2008; Han et al., 2019).

As mentioned above, high LMX aids in satisfying the competence and autonomy of employees and improves interpersonal relatedness. Based on self-determination theory, the fulfillment of these three needs will then improve the psychological health of employees. In their review of the antecedents of physical and mental health, Ryan and Deci (2001, p. 147) highlighted that when an individual can satisfy the three types of needs that are recognized in self-determination theory, the three elements of subjective well-being will increase (life satisfaction, high frequency of positive moods, and low frequency of negative moods). Baard et al. (2004) surveyed employees from two companies and found that when employees experienced high autonomy support from managers, this satisfied the employees’ needs for competence, autonomy, and relatedness, thereby improving their performance and psychological health (including lowering anxiety and depression).

Regarding the connection between positive mood and creative performance in employees, Dutton (2003) suggested that high-quality relationships help to stimulate positive moods, thereby improving creativity. George and Zhou (2007) also found that employees with positive moods are more likely to have divergent thoughts, more fluent thought composition, and better observational abilities. Therefore, these employees tend to connect disparate entities and improve their creative performance. Han et al. (2019) found that high-activity, positive moods, including happiness, concentration, and feeling active and interested, had significant positive correlations with creativity, while the low-activity, negative mood states of feeling tired and sleepy were associated with low creativity. Accordingly, we propose the following hypothesis:

**H2**: Positive mood is a mediating mechanism between LMX and creative performance. That is, high LMX will strengthen positive moods in employees, thereby encouraging them to exhibit greater creative performance.

## Materials and Methods

### Participants and Procedure

The participants of this study were employees and their immediate supervisors in township enterprises in the Pearl River Delta in China. The supervisors assessed the creative performance of their subordinates, while the employees responded to questions on LMX, intrinsic motivation, and positive moods. Questionnaires were used to collect data. In order to ensure that the employees would accurately match with their immediate supervisors, we coded all questionnaires and made it easy for leaders to distinguish different employees. To expand the diversity of the sample, we recruited individuals of different genders, industries, and departments. In addition, we entrusted individuals with strong influence in the organizations to distribute the questionnaires. Prior to distributing them, we conducted interviews with the employee and his/her immediate supervisor to determine their willingness to participate. For participants who elected to use electronic rather than paper questionnaires, email was used to provide a link to the questionnaires. In addition, participants were notified in the questionnaire instructions that the data would be used only for academic purposes and anonymized to protect personal privacy. The survey was conducted anonymously.

A total of 430 pairs of questionnaires were distributed, and 413 were returned. After screening for invalid and incomplete questionnaires, 386 pairs of valid questionnaires were finally obtained. The recovery rate of valid questionnaires was 89.8%. Most subordinate individuals were men (55.6%) and most participants were university or college graduates (73.3%). Employees had a mean age of 28.7 years, worked in their company for an average of 3.5 years, and been under their immediate supervisor for an average of 2.8 years. Manufacturing accounted for 37.5% of the sample and services accounted for 62.5%. In terms of departments, production and manufacturing accounted for 6.7%; R&D, 12.2%; operations, 37.9%; administrative management, 17.3%; and other departments, 25.9%.

### Measurement of Study Variables

#### Creative Performance

The 13-item scale developed by George and Zhou (2001) was completed by the supervisor to assess their subordinate’s creative performance. Example items include “He/she often has new and innovative ideas,” “He/she can come up with new and practical ideas to improve performance,” and “He/she often has a fresh approach to problems.” Responses are given on a six-point Likert-scale (score: 1–6 points), with the options ranging from “Extremely disagree” to “Extremely agree” (Cronbach’s α = 0.96).

#### Manager-Employee Exchange

The LMX 7 scale developed by Graen and Uhl-Bien (1995) was employed in this study to measure LMX. Example items include “I think my immediate superior understands my job problems and needs” and “I think my immediate superior recognizes my potential.” Responses are given on a five-point Likert-scale (Cronbach’s α = 0.85). The specific response options vary across questions (e.g., “Not at all” to “A great deal” or from “Not at all” to “Fully”).

#### Intrinsic Motivation

The 15-item Work Preference Inventory (WPI) developed by Amabile et al. (1994) was used to measure intrinsic motivation. Example items include “I want my work to provide me with opportunities for increasing my knowledge and skills” and “What matters most to me is enjoying what I do.” The responses are given on a six-point Likert-scale, with the options ranging from “Extremely disagree” to “Extremely agree” (Cronbach’s α = 0.86).

#### Positive Moods

The 10-item Positive and Negative Affect Schedule (PANAS) scale developed by Watson et al. (1988) was used to measure positive moods. Example items include “enthusiastic,” “determined,” and “inspired.” Responses are given on a five-point Likert-scale, with the options ranging from “Very slightly or not at all” to “Extremely.”

As positive moods in this study were considered to reflect individual mood states rather than traits, the instructions referenced a specific time-period (Watson et al., 1988). Further, the recommendation of George and Jones (1996) that the measurement of moods in employees should cover the preceding 2 weeks was used. Therefore, the participants were asked “To what extent did you feel this way within the last 2 weeks?” (Cronbach’s α = 0.87).

#### Control Variables

In this study, education level, work experience, and industry were control variables. Amabile (1983) suggested that employees’ knowledge or skills determine their problem-solving creativity, and their education level reflects their learned knowledge or skills. Therefore, education level was a control variable. Regarding work experience, the longer an employee works in a certain job, the more experience and effectiveness he/she will accumulate; these in turn will affect creative performance at work (Gong et al., 2009). Finally, previous studies have suggested that industry type may be associated with creative performance (Tierney and Farmer, 2002). Therefore, this study used industry type as a control variable.

## Results

### Descriptive Statistics and Correlations

Table 1 shows the means, standard deviations, and correlation coefficients of the variables in this study. There was a significant positive correlation of LMX with intrinsic motivation and positive mood (*r* = 0.38, *p* < 0.001; *r* = 0.39, *p* < 0.001, respectively). Similarly, intrinsic motivation and positive mood were significantly positively correlated with creative performance (*r* = 0.26, *p* < 0.001; *r* = 0.25, *p* < 0.001, respectively). In addition, the correlation between LMX and creative performance was significant (*r* = 0.18, *p* < 0.01).

**TABLE 1:** Means, standard deviations, and correlations between the study variables.

**Variable**	***M***	***SD***	**1**	**2**	**3**	**4**	**5**	**6**	**7**
1. Years of education	15.92	1.65	(–)						
2. Firm tenure	3.51	3.39	−0.19**	(–)					
3. Category of industry	0.33	0.48	0.07	0.02	(–)				
4. LMX	3.74	0.67	0.03	−0.03	0.01	(0.85)			
5. Intrinsic motivation	4.54	0.48	0.15*	−0.06	−0.01	0.38***	(0.86)		
6. Positive moods	3.36	0.66	0.13*	−0.11*	0.04	0.39***	0.47***	(0.87)	
7. Creative performance	4.01	0.93	−0.01	−0.02	−0.13*	0.18***	0.26***	0.25***	(0.96)

Internal consistency reliabilities are in parentheses. N = 386. *p < 0.05. **p < 0.01. ***p < 0.001. LMX, leader-member exchange.

### Measurement Model

Structural equation modeling (SEM) via LISREL 8.8 software was used to test the measurement model and validate the study’s hypotheses. Table 2 shows the confirmatory factor analysis results for the measurement model. Regarding convergent validity, the t-values of the factor loadings for all questions reached significance (*p* < 0.001). The composite reliability (CR) scores for the variables were all higher than 0.70 (LMX: CR = 0.83; intrinsic motivation: CR = 0.85; positive mood: CR = 0.85; creative performance: CR = 0.96); that is, all variables had good internal consistency. In terms of discriminant validity, we tested a theoretical model (Model 1) and five other models (Models 2–6). Model 1 exhibited better IFI, CFI, NNFI, and RMSEA values compared to the other models (Anderson and Gerbing, 1988). Regarding the discriminant validity of the variables, we calculated the confidence interval of the correlation coefficient between pairs of variables (Anderson and Gerbing, 1988) to determine discriminant validity. Bootstrapping with 1,000 replications was used with the hypothetical four-factor model to obtain 95% confidence intervals. If a confidence interval did not include the value 1, this indicated that discriminant validity was present between variables (Bagozzi and Phillips, 1982). The 95% confidence intervals of pairwise correlation coefficients between all variables were between 0.08 and 0.57, that is, none included the value 1. Therefore, the four-factor measurement model was considered acceptable.

**TABLE 2:** Results of confirmatory factor analysis of the study variables.

**Model**	**χ^2^**	***df***	**χ^2^/*df***	**△χ^2^**	**IFI**	**CFI**	**NNFI**	**RMSEA**
Null model	29033.21	1041	27.88	–	–	–	–	–
1. Four-factor	2951.28	988	2.99	–	0.94	0.94	0.94	0.08
2. Three-factor	3754.43	989	3.80	801.31	0.93	0.93	0.93	0.09
3. Three-factor	3763.57	989	3.81	815.81	0.93	0.93	0.93	0.09
4. Three-factor	3819.61	989	3.86	857.51	0.93	0.93	0.93	0.09
5. Two-factor	4548.75	991	4.59	1597.20	0.92	0.92	0.91	0.10
6. One-factor	11521.32	994	11.59	8563.41	0.84	0.84	0.84	0.18

*N = 386. Model 1 (four-factor model) is the theoretical model that included all of the study variables (LMX, intrinsic motivation, positive moods, creative performance) as four independent factors. Model 2 (three-factor model) combined LMX and intrinsic motivation as one factor and treated the other two variables as two independent factors. Model 3 (three-factor model) combined LMX and positive moods as one factor and considered the other two variables as two independent factors. Model 4 (three-factor model) combined intrinsic motivation and positive moods as one factor and considered the other two variables as two independent factors. Model 5 (two-factor model) combined LMX, intrinsic motivation, and positive moods as one factor, and creative performance as an independent factor. Model 6 (one-factor model) combined all four variables as one factor. IFI, incremental fit index; CFI, comparative fit index; NNFI, non-normed fit index; RMSEA, root mean square error of approximation.*

### Validation of the Theoretical Model and Study Hypotheses

Table 3 shows the validation of the theoretical model and study hypotheses. Apart from the theoretical model (Model 1), we also tested a nested model (Model 2) that increased the direct effect of LMX on creative performance to assess the possibility of partial mediation. As shown in Table 3, when the direct effect of LMX on creative performance was added to Model 2 (△χ^2^ = −2.29), the χ^2^-value of Model 2 was smaller than that of Model 1. However, the explained variance of the overall model did not reach significance when the direct path from LMX to creative performance was added. Therefore, we did not include the path from LMX to creative performance. Next, we compared the alternative models that were nested within the theoretical model (Models 3–5). The IFI, CFI, and NNFI values of Models 3–5 were acceptable (>0.90) and RMSEA was lower than 0.10. Therefore, we used the method described by Wang et al. (2005) to further compare the standardized path coefficients and significance levels of variables in the theoretical and alternative models to determine the final model. Regarding the standardized path coefficients, all paths in the theoretical model reached significance. In Model 3, all paths were significant except the direct path from LMX to creative performance. Therefore, the theoretical model was superior to Model 3.

**TABLE 3:** Summary of model fit indices.

**Model test**	**χ^2^**	***df***	**χ^2^/*df***	**△χ^2^**	**IFI**	**CFI**	**NNFI**	**RMSEA**
1	3267.78	1125	2.90	–	0.94	0.94	0.94	0.08
2	3265.49	1124	2.90	−2.29	0.94	0.94	0.94	0.08
3	3163.99	1125	2.81	–	0.95	0.95	0.94	0.08
4	3170.41	1125	2.82	–	0.95	0.95	0.94	0.08
5	3162.59	1122	2.82	–	0.95	0.95	0.94	0.08

*N = 386. Model 1 is the theoretical model. Model 2 used the theoretical model as its basis and added the direct path from the independent variable to dependent variables. Model 3 used positive mood as an independent variable; the mediator variables were intrinsic motivation and leader-member exchange (LMX) and the dependent variable was creative performance. Model 4 used intrinsic motivation as an independent variable, positive mood and LMX as mediator variables, and creative performance as the dependent variable. In Model 5, intrinsic motivation and positive mood were the independent variables, LMX was the mediator variable, and the dependent variable was creative performance. IFI, incremental fit index; CFI, comparative fit index; NNFI, non-normed fit index; RMSEA, root mean square error of approximation.*

In Model 4, the path coefficient of intrinsic motivation to LMX was 0.46 (*p* < 0.001), which was lower than that of the theoretical model (0.48, *p* < 0.001), while the path coefficient of positive mood to creative performance was 0.18 (*p* < 0.001), which was higher than that of the theoretical model (0.13, *p* < 0.05). Although the path coefficients of the two models suggested better and worse performance for specific paths, self-determination theory emphasizes the fulfillment of needs by interactions between individuals and the social situation. Therefore, the fulfillment of the competence, autonomy, and relatedness needs of employees due to LMX was used to explain the increased intrinsic motivation of employees (i.e., attitudinal processes). Furthermore, according to Reis et al. (2000) and Ryan and Deci (2001), who found that relatedness can aid in the generation of positive moods (emotional process), it is more appropriate to consider LMX as an independent variable, while intrinsic motivation and positive mood should be considered as mediating mechanisms. Therefore, with reference to extant theory, the theoretical model was superior to Model 4.

**FIGURE 1:**
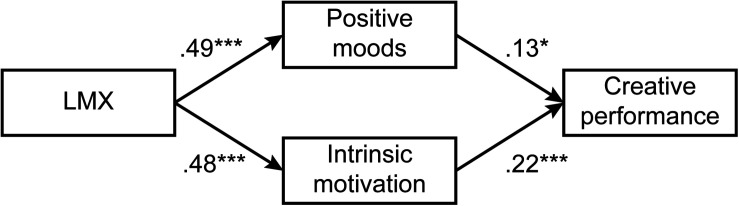
Structural equation model with moderation results. *p < 0.05, ***p < 0.001.

Regarding Model 5, its path coefficients were significant but smaller than those of the theoretical model for intrinsic motivation toward LMX (*r* = 0.28, *p* < 0.001) and for positive mood toward LMX (*r* = 0.31, *p* < 0.001; theoretical model: LMX to intrinsic motivation, *r* = 0.48, *p* < 0.001; LMX to positive mood: *r* = 0.49, *p* < 0.001). Therefore, we considered the theoretical model to be the final model. The path diagram and standardized path coefficients are shown in Figure 1.

To validate indirect effects, we used bootstrapping with 1,000 samples to calculate the indirect effects of intrinsic motivation and positive mood and their bias-corrected 95% confidence intervals. When the confidence interval does not include zero, this denotes a significant indirect effect (Preacher and Hayes, 2004). Based on the bootstrapping results, the indirect effect of intrinsic motivation was 0.12 and bias-corrected 95% confidence interval did not include 0 (CI: 0.05–0.16). Thus, the indirect effect was significant, and Hypothesis 1 was supported. The indirect effect of positive mood was 0.08 and the bias-corrected 95% confidence interval did not include 0 (CI: 0.04–0.17). Therefore, Hypothesis 2 was supported.

## Discussion

### Theoretical Contributions

Our study makes several theoretical contributions. First, we found that high LMX can stimulate intrinsic motivation in followers, thereby improving their creative performance. We employed self-determination theory and illustrated that in a high LMX environment, the supervisor will provide more support resources to employees, such as more information, informal feedback, and resources required to complete tasks. This can satisfy the competence needs of employees and lead them to believe that they are able to complete these tasks with the support that they have available. In contrast to low LMX environments, employees with high LMX are empowered by their managers, which also fulfills the autonomy needs of employees. In addition to satisfying competence and autonomy needs, high LMX provides greater security and relatedness in interpersonal relationships, thereby increasing intrinsic motivation toward work and promoting creative performance. This is consistent with Amabile (1996), who suggested that intrinsic motivation is an important mechanism in how situations affect creativity.

Second, based on self-determination theory, we propose that high LMX will provide more psychological and emotional support, such as encouragement and care. This addresses the relatedness needs of individuals in terms of interpersonal emotion. When the three needs of competence, autonomy, and relatedness are satisfied in employees, they will tend to show positive emotions, which leads to cognitive models that promote increased diversity of thought and are conducive to the generation of creative performance. The results of the current study are consistent with those of George and Zhou (2007); Baas et al. (2008), and Han et al. (2019), who studied the relationship between positive mood and creative performance.

Third, previous studies that examined the mediating mechanisms between LMX and creativity focused on variables such as felt obligation and psychological empowerment (Pan et al., 2012), self-efficacy (Liao et al., 2010), and feelings of energy (Atwater and Carmeli, 2009; Adil and Awais, 2016). These mediating mechanisms are based on social exchange theory or social cognitive theory and represent employee responses regarding perceived LMX. However, according to Amabile’s (1983) componential theory of creativity, the effector path of manager support to employee creativity promotes the intrinsic motivation of followers toward creative problems, in addition to assisting in project planning and professional skill development (Amabile et al., 2004). Furthermore, Dutton (2003) indicated that high-quality relationships aid in stimulating positive moods, thereby improving individual creativity. In contrast to previous mediator studies, our study used self-determination theory as the basis when examining the mediating mechanisms between LMX and creative performance. We illustrated that managers can satisfy the need for supportive work resources (needs for self-competence and autonomy) and psychological and emotional aspects (relatedness needs) in a high LMX environment, which guides and stimulates intrinsic motivation and positive moods in employees, thereby improving creative performance.

Fourth, Atwater and Carmeli (2009) and Adil and Awais (2016) found that feelings of energy mediate the relationship between LMX and creativity. However, they used self-assessment for all variables, and thus there might have existed common-method variance. Our study addressed this issue by distinguishing the source of creative performance from other variables. The employees’ creative performance was assessed by their supervisors. This simultaneously avoided overestimation of creative performance in employees due to excessively positive self-perceptions and ensured the authenticity of the results.

Finally, we used self-determination theory to explain the mediating effects of intrinsic motivation and positive moods. Our study extends self-determination theory to explain how external factors (such as LMX) can develop intrinsic motivation and increase positive moods (the antecedents) and further expands this theory to individual behavioral performance (the outcomes). At the same time, we integrated the effects of LMX on creative performance through emotional and attitudinal mechanisms. This provides a perspective for further understanding the relationship between LMX and creative performance.

### Implications for Management

Our study has practical implications as well. Managers can create a reliable and relaxed environment by establishing high LMX and improving employee creativity. That is, managers have considerable psychological influence on employees. Therefore, if an organization wants to encourage creativity, it could begin by training managers to demonstrate high LMX by strengthening their relationship skills. This would provide an environment that fosters creativity in employees.

The results also indicated that psychological resources that promote creative performance include intrinsic motivation and positive mood. Regarding intrinsic motivation, leaders must focus on the cultivation and adoption of encouragement techniques and strategies. In a high LMX environment, leaders can appropriately provide assistance through clear feedback and specific supportive behaviors, such as by giving employees the opportunity and right to participate in corporate affairs and treating subordinates with respect and kindness, so that employee needs for competence, autonomy, and relatedness are sufficiently fulfilled. A positive mood can be considered as an indicator of employees adjusting their cognitive model. Therefore, leaders should establish a joyful and encouraging atmosphere to enhance the positive mood of their employees. In addition, leaders can show concern, make an effort to understand employees’ difficulties and needs, and provide work-related feedback to increase employees’ feelings of being valued in the organization. As for the employees, cooperating with their managers actively will not only help in satisfying the requirements of self-determination theory but also accelerate the speed and improve the effectiveness of communication exchange; hence, it will help to create an atmosphere suitable for innovation by fostering trust and communication.

### Study Limitations and Future Research Suggestions

This study has the following limitations: First, a cross-sectional study design was employed, which limits attributions of causality (Podsakoff et al., 2003; Dulebohn et al., 2012). However, this study measured positive mood as a state rather than as a trait. Therefore, participants answered questions over a period of 2 weeks to ensure that any measured positive mood was a state. Moreover, although many studies have used the PANAS scale to measure individuals’ positive emotional state (e.g., Craske et al., 2019; Haga et al., 2020; Plys and Desrichard, 2020), there are still some limitations. For example, individuals may be influenced by the need for social approval and, thus, exaggerate their positive mood. Therefore, future researchers can use the implicit measurement method to measure individuals’ mood. In addition, brain science methods (e.g., electroencephalography) can also be used to measure the degree of brain lateralization, to more accurately reflect the individual’s emotional state, since studies have shown that a positive mood is left-lateralized and a negative mood is right-lateralized (Helmstaedter et al., 2004; Kaiser and Wyczesany, 2008; Butler et al., 2018). In addition, as described by George and Zhou (2002), creative performance is a continuously occurring organizational behavior that may occur for a duration of longer than 2 weeks. Therefore, we should expand the time-frame in which creative performance was measured (George and Zhou, 2002).

Regarding questionnaire design, the suggestions of Podsakoff et al. (2003) were used in this study and the sequence of questionnaires given to employees followed the order of “mediator variables in front and independent variables behind” to avoid the possibility of causality confusion. In addition, according to self-determination theory, the external environment will satisfy competence, autonomy, and relatedness, which will ultimately lead to behavioral changes. Specifically, relatedness will engender positive moods, while competence and autonomy will increase intrinsic motivation, ultimately leading to creative performance. Therefore, it is most appropriate to consider LMX, positive mood, and intrinsic motivation as antecedents of creative performance.

The questionnaires in this study instructed participants to provide answers based on their mood during the prior 2 weeks; however, the assessment of creative performance by their supervisors might not have corresponded to the time-frame of self-assessment. This is because employees usually undertake many tasks simultaneously and creative performance occurs continuously. Therefore, the time-period limitation might have resulted in assessment limitations.

Finally, the three types of psychological needs (competence, autonomy, and relatedness) in self-determination theory were used to describe how LMX can satisfy these three needs to promote the development of intrinsic motivation, and ultimately lead to creative performance. To measure intrinsic motivation, we used the WPI (Amabile et al., 1994). Although Amabile et al. included two needs (competence and autonomy) when designing this scale, they failed to account for relatedness. Therefore, although this study found that these three factors could promote the development of intrinsic motivation, the scales may not completely correspond to the intrinsic motivation caused by the three needs, which represents a limitation of this study. In contrast, Kasser et al. (1992) and Deci et al. (2001) used the Basic Need Satisfaction Scale to measure individuals’ satisfaction with the three needs. However, our study found that this scale measured the degree of satisfaction with the three needs but not individual needs. Therefore, if the Basic Need Satisfaction Scale were used to the replace measurement of intrinsic motivation, it would not completely reflect the concept of intrinsic motivation. It is recommended that future studies simultaneously include the measurement of basic needs satisfaction and intrinsic motivation to reflect the process of need satisfaction in the promotion of intrinsic motivation, as per self-determination theory.

As this study broadly examined creative performance for different types of work, we did not limit the type of work in which participants were employed. In future studies, the type of work could be clearly delineated to test whether there are differences in creative performance processes between different types of work. In addition, one could also precisely examine whether LMX can improve the creative performance of different types of employees through the same process.

Besides intrinsic motivation and positive mood, future studies should also examine other possible mediator variables, such as self-efficacy and goal commitment of employees; further, different processes could be used to examine the relationship between LMX and creative performance. In addition, regulating variables of the mediating process between LMX and creative performance could be explored. For example, does power distance or psychological safety affect employee satisfaction with respect to competence and autonomy? Moreover, according to self-determination theory, an individual’s self-adjustment to a situation determines their intrinsic motivation toward events (Ryan and Deci, 2000). Therefore, we recommend that future studies employ self-concept as a moderator variable to examine its effects on the relationship between LMX and intrinsic motivation.

This was a quantitative study. In the future, qualitative methods could be used by employing participant observation or interviews. Examples include focus group interviews and open-ended questionnaires. Such approaches could promote a deeper understanding of the relationship between LMX and creative performance and its mediating processes.

## Conclusion

This study investigated two mediating processes from LMX to creative performance and found that: (1) in attitudinal processes, high LMX will improve the creative performance of followers by stimulating their intrinsic motivation; and (2) in emotional processes, high LMX will improve employee creativity by enhancing their positive mood.

## Data Availability Statement

The raw data supporting the conclusions of this article will be made available by the authors, without undue reservation.

## Ethics Statement

This study was carried out in accordance with the recommendations of the Hainan University Research Ethics Committee. The protocol was approved by the Hainan University Institutional Review Board (approval number: 201901335). The patients/participants provided their written informed consent to participate in this study.

## Author Contributions

ZX and AF substantially contributed to the conception, the design of the work as well as the preparation of the draft. NW and TY contributed to the analysis and interpretation of the data. JJ and GH critically reviewed and contributed important intellectual input. All authors contributed to the article and approved the submitted version.

## Conflict of Interest

The authors declare that the research was conducted in the absence of any commercial or financial relationships that could be construed as a potential conflict of interest.
